# Caries Increment and Salivary Microbiome during University Life: A Prospective Cohort Study

**DOI:** 10.3390/ijerph17103713

**Published:** 2020-05-25

**Authors:** Yoko Uchida-Fukuhara, Daisuke Ekuni, Md Monirul Islam, Kota Kataoka, Ayano Taniguchi-Tabata, Daiki Fukuhara, Naoki Toyama, Terumasa Kobayashi, Kohei Fujimori, Nanami Sawada, Yoshiaki Iwasaki, Manabu Morita

**Affiliations:** 1 Department of Preventive Dentistry, Okayama University Hospital, Okayama 700-8558, Japan; de19026@s.okayama-u.ac.jp (A.T.-T.); de20041@s.okayama-u.ac.jp (D.F.); 2 Advanced Research Center for Oral and Craniofacial Sciences, Okayama University Dental School, 2-5-1 Shikata-cho, Kita-ku, Okayama 700-8558, Japan; de18017@s.okayama-u.ac.jp; 3 Department of Oral Morphology, Graduate School of Medicine, Dentistry and Pharmaceutical Sciences, Okayama University, Okayama 700-8558, Japan; 4 Department of Preventive Dentistry, Okayama University Graduate School of Medicine, Dentistry and Pharmaceutical Sciences, Okayama 700-8558, Japan; dekuni7@md.okayama-u.ac.jp (D.E.); p3a99o50@s.okayama-u.ac.jp (M.M.I.); pu171qxi@s.okayama-u.ac.jp (N.T.); de421015@s.okayama-u.ac.jp (T.K.); k-fujimori@okayama-u.ac.jp (K.F.); de422027@s.okayama-u.ac.jp (N.S.); mmorita@md.okayama-u.ac.jp (M.M.); 5 Health Service Center, Okayama University, Okayama 700-8530, Japan; yiwasaki@okayama-u.ac.jp

**Keywords:** salivary microbiome, sequence analysis, young adult, dental caries, saliva, oral health

## Abstract

The purpose of this 3-year prospective cohort study was to explore the relationship between an increase in dental caries and oral microbiome among Japanese university students. We analyzed 487 students who volunteered to receive oral examinations and answer baseline (2013) and follow-up (2016) questionnaires. Of these students, salivary samples were randomly collected from 55 students at follow-up and analyzed using next-generation sequencing. Students were divided into two groups: increased group (Δdecayed, missing, and filled teeth (ΔDMFT) score increased during the 3-year period) and non-increased group (ΔDMFT did not increase). Thirteen phyla, 21 classes, 32 orders, 48 families, 72 genera, and 156 species were identified. Microbial diversity in the increased group (*n* = 14) was similar to that in the non-increased group (*n* = 41). Relative abundances of the family *Prevotellaceae* (*p* = 0.007) and genera *Alloprevotella* (*p* = 0.007) and *Dialister* (*p* = 0.039) were enriched in the increased group compared with the non-increased group. Some bacterial taxonomic clades were differentially present between the two groups. These results may contribute to the development of new dental caries prevention strategies, including the development of detection kits and enlightenment activities for these bacteria.

## 1. Introduction

Dental caries caused by some pathogens of the oral microbiome is a widespread disease. In the Global Burden of Disease 2015 Study, untreated caries in permanent teeth was the most prevalent condition, affecting 2.5 billion people worldwide [1]. The cause of dental caries is multifactorial. In addition to major microbial species mutans streptococci (predominantly *Streptococcus mutans* and *S. sobrinus*), physical, biological, environmental, behavioral, and lifestyle-related factors are risk factors for dental caries [2].

Recently, next-generation DNA sequencing (NGS) analyses have attracted attention for use in microbiome studies, including dental caries. A pyrosequencing (NGS analysis) study revealed genera *Prevotella*, *Lactobacillus*, and *Streptococcus* were increased in carious dentin compared with healthy dentin [3]. Another study revealed that genera *Alloprevotella*, *Atopobium*, *Lautropia*, *Megasphaera*, *Selenomonas*, and *Veillonella* were enriched in saliva from a high-risk caries group [4]. According to the ecological hypothesis of dental caries [5], the proportion of acidogenic and aciduric taxa, such as mutans streptococci and *Lactobacillus*, are increased and lead to demineralization. In addition, this hypothesis suggests that amino acid-degrading microbial taxa, including *Prevotella*, and *Fusobacterium* species may also cause the acidic environment required for demineralization [5,6]. These results indicate that both mutans and non-mutans streptococci may contribute to dental caries. However, many caries studies using sequencing analyses targeted young children with primary dentition, and few studies have focused on young adults and older people [4,7,8,9,10]. Whether a relationship between the increase in dental caries and oral microbiome exists in these latter populations remains unclear.

In Japan, there remains a high caries prevalence in young adults. Among young adults (20–24 years), 79.4% had caries experience [11]. Thus, it is crucial to prevent dental caries in the general population.

Therefore, we hypothesized that an increase in dental caries during university life is associated with a unique oral microbiome. The purpose of this 3-year prospective cohort study was to explore the relationship between an increase in caries and oral microbiome among Japanese university students. In addition, we investigated the association between other factors and caries increment.

## 2. Materials and Methods

### 2.1. Study Population

The inclusion criteria were Japanese students who volunteered to receive oral examinations at the Health Service Center of Okayama University both in April 2013 (baseline) and April 2016 (follow-up). We excluded students who provided incomplete responses in their questionnaires.

### 2.2. Ethical Procedures and Informed Consent

All study protocols were approved by the ethics committee of Okayama University Graduate School of Medicine, Dentistry, and Pharmaceutical Sciences and Okayama University Hospital (no. 1060). All targeted participants gave their informed verbal consent for study participation. This study followed the strengthening the reporting of observational Studies in Epidemiology (STROBE) guidelines.

### 2.3. Questionnaire

At baseline, students answered questions concerning age, sex, systemic diseases, and oral health behaviors (daily frequency of tooth brushing, use of dental floss, and visits to dental clinics for regular checkups) [12]. Furthermore, at follow-up, four additional questions were included about fluoride dentifrices, knowledge of the effectiveness of fluoride, daily frequency of eating sweets, and smoking during university life.

### 2.4. Oral Examination

Oral examinations were performed by five calibrated dentists (Daisuke Ekuni, Kota Kataoka, Mayu Yamane-Takeuchi, Shinsuke Mizutani, and Tetsuji Azuma). After counting the number of teeth, the oral hygiene state was evaluated using the Debris Index-Simplified (DI-S) score [13]. The decayed, missing, and filled teeth (DMFT) scores was recorded according to the World Health Organization criteria [14]. After the theoretical training, to assess intra- and inter-examiner agreement, DMFT scores were recorded and repeated within a 2-week interval in two volunteers. Data were analyzed using a non-parametric kappa test. The kappa values were >0.8.

### 2.5. DNA Extraction and NGS Analysis

At follow-up, unstimulated saliva (>1 mL) was randomly collected into sterile ice-chilled 15 mL tubes from students (from 09:00 to 16:00) before the dental examination and frozen at −80 °C until analysis. To prevent possible protein dilution, unstimulated saliva was collected instead of stimulated saliva [15,16,17]. A random number list was used for selecting participants for saliva collection. The saliva of selected participants was not collected at baseline. Saliva DNA was collected using the QIAamp DNA Mini Kit (Qiagen, Hilden, Germany) according to the manufacturer’s instructions using sterile equipment and DNA removal reagents for laboratory instruments. Collected DNA was stored at −20 °C for further analysis. For amplified bacterial DNA, V3 and V4 regions of the 16S rRNA gene were amplified using primers 357F (5′-TCGTCGGCAGCGTCAGATGTGTATAAGAGACAGCCTACGGGNGGCWGCAG-3′) and 781R (5′-GTCTCGTGGGCTCGGAGATGTGTATAAGAGACAGGACTACHVGGGTATCTAATCC-3′) at Okayama University Hospital Biobank (Okayama University Hospital, Okayama, Japan) according to the standard protocol using the MiSeq platform (MiSeq Reagent V3 600 cycles, Illumina, San Diego, CA, USA). Quality of raw sequence reads was checked using FastQC (version 0.11.3, Babraham Bioinformatics, Cambridge, UK) and analyzed using USEARCH (version 8.0.1623, https://www.drive5.com/usearch/) at the Oral Microbiome Center (Taniguchi Dental Clinic, Kagawa, Japan). After removing chimeric reads, duplicated reads, and short reads <400 bp, preprocessed reads of each sample were clustered into operational taxonomic units (OTUs) at 97% level of nucleotide similarity using UCLUST algorithm to determine the number of OTUs. Furthermore, these reads were analyzed to identify human oral taxa using the Human Oral Microbiome Database (version 14.5; http://www.homd.org/).

### 2.6. Statistical Analyses

We estimated the sample size for the saliva examination based on a previous study [4] using G*Power (version 3.1.9.4, Düsseldorf, Germany). The difference in α diversity (Simpson index; caries group: 0.075 ± 0.019, healthy group: 0.099 ± 0.037) was selected as the primary outcome [4]. To calculate the effect size, mean and standard deviation of 0.024 ± 0.018 was considered to detect a difference in α diversity between the two communities. Based on the data, the minimum sample size required was 52 to provide a power of 91% with an alpha of 0.05 by *t*-test. Participants were divided into two groups based on the change in DMFT score during the 3-year follow-up period; participants with ΔDMFT > 0 were categorized into the “increased group,” while participants with ΔDMFT = 0 were categorized into the “non-increased group” [18]. The normality of the data was confirmed by the histogram and quantile-quantile plot. The Mann–Whitney U test and Fisher’s exact test were used to determine the presence of significant differences in variables of oral examination and questionnaire between increased and non-increased groups. Associations between variables and dental caries were examined in a series of logistic regression models, and the odds ratio (OR) and 95% confidence interval (CI) were calculated. Logistic regression models were reviewed for goodness-of-fit and validated using the Hosmer–Lemeshow statistic. A *p*-value < 0.05 was considered significant. Statistical analyses were performed using SPSS (version 25.0; IBM, Tokyo, Japan).

Alpha diversity was determined using R (version 3.4.3; The R Project for Statistical Computing, http://www.R-project.org). The species richness of saliva microbiota of individuals was measured by Chao1 and the abundance-based coverage estimator (ACE) indices. The diversity of the saliva microbiota was measured by Shannon and Simpson indices. Microbial beta diversity was visualized by principal coordinates analysis using the Calypso software tool (http://bioinfo.qimr.edu.au/calypso/). The Mann–Whitney U test was used to assess significant differences in the abundance of taxa between increased and non-increased groups. Rarefaction curves were calculated using Calypso to compare microbial richness among samples. To compare the microbial composition between increased and non-increased groups, relative abundances (%) were calculated from the taxonomic abundance count divided by preprocessed reads. Linear discriminant analysis (LDA) effect size (LEfSe) methods were used to identify taxa with differentiating relative abundance using the online interface Galaxy (http://huttenhower.sph.harvard.edu/lefse/). The threshold for the logarithmic LDA score for biomarker discovery was set at 2.0 [19,20].

## 3. Results

### 3.1. Participant Characteristics

**Figure 1:**
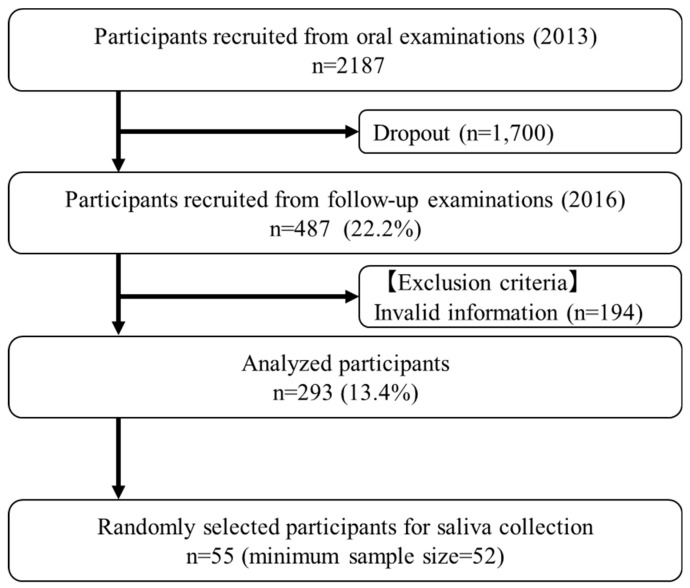
Flowchart of study participants. This flowchart shows the process for selecting analyzed participants. At baseline, 2187 students received the oral examination. Because follow-up oral examination was not mandatory, only 487 students received a second oral examination. Of these students, 55 students were randomized and consented to saliva collection.

The study flowchart is shown in Figure 1; 293 students (follow-up rate: 13.4%; 149 males, 144 females; mean age and standard deviation at baseline: 18.2 ± 0.77 years) were analyzed (Table 1). At follow-up, the DI-S score of selected students (*n* = 55) was significantly lower than that of all participants (*n* = 293; *p* < 0.05, *t*-test). No significant differences were observed in the other parameters evaluated between selected students and all participants (*p* < 0.05, Mann–Whitney U test and Fisher’s exact test).

In all participants, DMFT scores, DI-S score at baseline, and the percentage of students who had knowledge of fluoride at follow-up were significantly higher in the increased group than those in the non-increased group (*p* < 0.05; Table 2). In the logistic regression analysis, increase in dental caries was significantly related to DMFT score (OR: 1.191, 95% CI: 1.033–1.373, *p* = 0.016) and DI-S score (OR: 3.6, 95% CI: 1.355–6.913, *p* = 0.007) at baseline.

### 3.2. Salivary Microbiome Analysis

**Figure 2:**
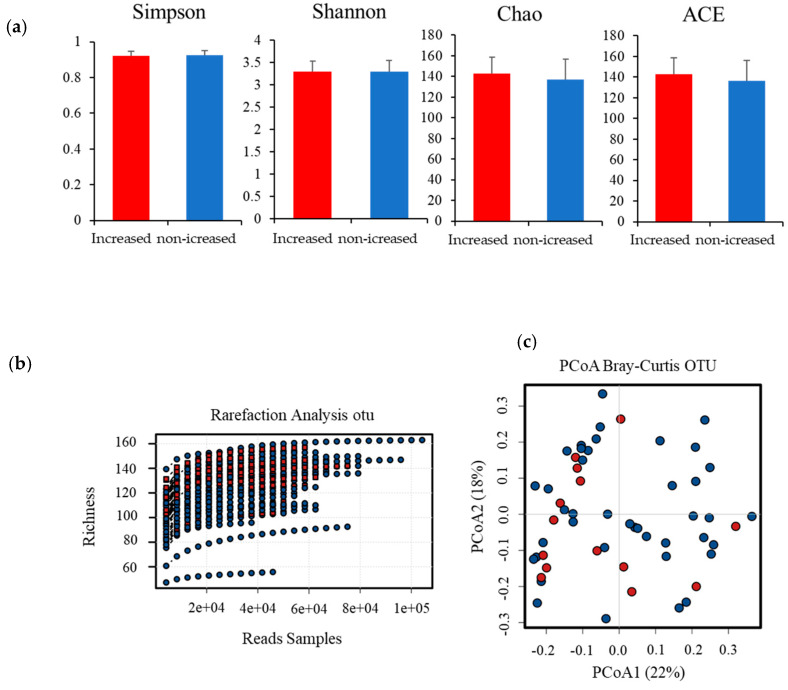
Comparison of the oral microbiome diversity between increased and non-increased groups. (**a**) Saliva microbiome diversity between increased (red) and non-increased groups (blue) was compared. Alpha diversity metrics for Simpson, Shannon, Chao1, and ACE indices were calculated and illustrated by box plots. There were no significant differences in diversity between increased and non-increased groups (*t*-test, *p* *≥* 0.05). (**b**) Rarefaction curves of increased (red) and non-increased (blue) groups based on the observed operational taxonomic unit (OTU). There were no significant differences in species richness between increased and non-increased groups (*t*-test, *p* ≥ 0.05). (**c**) Principal coordinates analysis (PCoA) based on the Bray–Curtis index. Increased (red) and non-increased (blue) groups did not tend to separate.

In our study, 3,195,127 quality-filtering reads (58,093 ± 16,532) from 55 saliva samples were used for analysis. A total of 196 OTUs were obtained from saliva samples. Of these, 13 phyla, 21 classes, 32 orders, 48 families, 72 genera, and 156 species were identified. There were no significant differences in the number of each taxonomic level between increased and non-increased groups (*p* ≥ 0.05). Furthermore, there were no significant differences in the species richness of individuals between increased and non-increased groups (Figure 2a; *p* ≥ 0.05). Moreover, no significant differences were observed in species diversity and bacterial communities between the two groups (Figure 2b,c; *p* ≥ 0.05).

### 3.3. Microbial Composition

**Figure 3:**
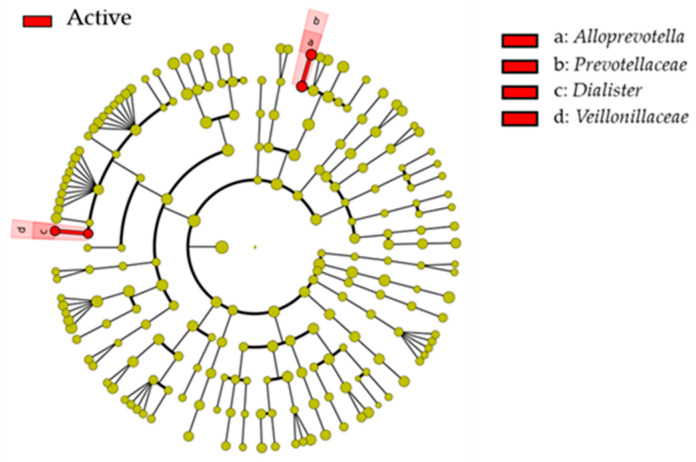
Linear discriminant analysis effect size (LEfSe) analysis to identify unique taxa associated with caries. Taxa at the family level were analyzed. Red areas of the cladogram were enriched in the increased group compared with the non-increased group. Linear discriminant analysis (LDA) scores ≥ 3.0 are shown.

There were no significant differences in microbial composition at class and order levels between increased and non-increased groups. At the phylum level, the increased group had a higher population of *Proteobacteria* than the non-increased group (Table 3; *p* = 0.029). At the family level, the increased group had a higher population of *Prevotellaceae* than the non-increased group (Table 3; *p* = 0.007). At the genus level, the increased group had a higher population of *Actinobaculum*, *Dialister*, and *Alloprevotella* than the non-increased group (Table 3; *p* < 0.05). At the species level, the increased group had higher populations of *Neisseria sicca*, *Alloprevotella* sp., *Dialister invisus*, *Cardiobacterium hominis*, *Acinetobacter* sp., *Gracilibacteria* (*GN02) [G-1]*, *Neisseria elongate*, *Actinomyces graevenitzii*, *Anaerolineae [G-1]*, *Dialister pneumosintes*, *Haemophilus haemolyticus*, *Actinobaculum* sp., *Corynebacterium matruchotii*, *Prevotella pleuritidis*, and *Neisseria sp*. than those in the non-increased group (Table 3; *p* < 0.05). Low percentages of *S. mutans* in saliva were detected in both groups. In LEfSe analysis, *Prevotellaceae* and *Veillonellaceae* at the family level and *Alloprevotella* and *Dialister* at the genus level were enriched in the increased group compared with the non-increased group (Figure 3).

## 4. Discussion

Various groups have provided information about the relationship between dental caries and the oral microbiome [4,7,8,9,10,21,22,23,24,25]. However, there is less information about this relationship in young adults or university students. In the present 3-year cohort study, the abundance of several taxa was significantly higher in the increased group than that in the non-increased group. In addition, poor oral hygiene and caries experience at baseline were significantly associated with an increase in dental caries during university life.

In this study, the observed taxa number was not significantly different between increased and non-increased groups. Moreover, the microbial structure was similar between the two groups. Jiang et al. reported that the microbial composition was similar between caries progression and non-progression groups among older people [23]. The findings from our study support these results.

The percentages of several taxa were significantly higher in the increased group than those in the non-increased group. In particular, further analysis using LEfSe methods revealed families *Prevotellaceae* and *Veillonellaceae* and genera *Alloprevotella* and *Dialister* were significantly enriched in the increased group compared with the non-increased group (LDA scores ≥ 3.0). *Prevotellaceae* was detected in carious dentin or saliva of participants with caries progression [24,25]. Furthermore, Eriksson et al. also reported that *Veillonellaceae* and *Dialister* were enriched in the saliva of patients with caries progression using the LEfSe method [20]. *Alloprevotella* was detected in the saliva of children with caries progression using LEfSe [4]. Using non-culture methods, non-mutans streptococci have been detected in carious dentin or saliva [26]. Furthermore, *Prevotellaceae* and *Veillonellaceae* species are known to produce acid [5,27]. These results indicate that several acid-producing bacteria or non-mutans streptococci may be associated with caries progression, which suggests new targets for preventing caries progression.

In this study, there were no significant differences in microbiome diversities between increased and non-increased groups. Previous studies exploring the oral microbiome to elucidate potential targets for caries prevention mainly targeted children with deciduous dental plaque or saliva. For example, a 2-year cohort study revealed that microbial diversity in saliva did not differ between caries progression and non-progression groups [10]. However, some cohort studies reported that salivary microbiome diversity was significantly different between participants with increased dental caries compared with those with non-increased dental caries [7,21,22]. According to these reports, whether salivary microbiome diversity is associated with caries progression remains controversial. Furthermore, few studies included young adults as subjects. A cross-sectional study exploring salivary microbiomes among families, including young adults, reported that dental caries did not impact microbial diversity [28]. While our findings support the results of this study, further cohort studies are required to investigate the relationship between microbiome diversity and an increase in dental caries.

Oral condition is also associated with caries progression. In a cohort study of adult participants in Sweden, DMFT scores of the caries progression group were higher than those in the non-progression group at baseline [29]. In addition, a previous study reported that the increased caries group had worse oral hygiene than that of the non-increased caries group [30]. Here, we demonstrated that DMFT and DI-S scores were associated with an increase in dental caries in the logistic regression analysis, supporting the results of these previous studies.

In this study, a low abundance of mutans streptococci was detected among the two groups. Mutans streptococci are traditionally recognized as the most common cause of dental caries [31]. Several studies also reported that there was no difference in the abundance of these species between caries-active and caries-free groups [7,8,9,32]. Furthermore, Takahashi and Nyvad proposed the “Ecological Plaque Hypothesis,” wherein acid-producing bacteria, excluding mutans streptococci, are also considered to lead to an imbalance in mineralization [26]. However, we did not investigate the microbiome in dental plaques of participants with caries. Further studies are therefore required.

In this study, we used saliva samples for microbiome analysis. Saliva collection is non-invasive, simple, and effective for mass examination. Some studies reported that plaque bacteria are released into saliva [9,33], and saliva provides a niche for both anaerobic and aerobic bacteria [34]. Furthermore, saliva is a biomarker that reflects oral health and systemic condition [35,36]. Thus, saliva evaluation has several advantages, especially in large-scale epidemiological studies or caries screening.

Our study has some limitations. First, there may have been selection bias, given the low follow-up rate (13.4%). In addition, the number of participants who had regular dental checkups at baseline and DI-S scores at follow-up differed between selected participants and total participants. However, increased dental caries was not related to these variables in the logistic regression analysis. Therefore, the selection bias might be small in this study. Second, all participants were recruited from among students who attended Okayama University. Therefore, our results may not be generalizable. Third, we could not investigate potential confounders, such as social capital [37,38] or socioeconomic status [39]. Fourth, we did not use a specific primer and probe for detecting *S. mutans*. Using the Human Oral Microbiome Database, we could detect the taxa using obtained sequencing reads. However, a previous study mentioned that V1–V2 or V3–V4 hypervariable regions (approximately 400–460 bp) is the limit for identifying detailed level of taxa [19]. Therefore, a wider region or specific primer is needed [19]. Fifth, we did not set a specific time for saliva collection; that is, saliva was collected from 09:00 to 16:00. However, we randomly selected participants and collected saliva at the last step of the general health examination. Therefore, we do not consider that eating or drinking affected salivary analysis. Finally, we could not collect saliva at baseline; however, we checked caries increment using DMFT at baseline and follow-up. Although Belstrøm et al. reported the stable condition of the salivary microbiome [40], we cannot rule out the possibility of changes in the salivary microbiome during the 3-year study period.

## 5. Conclusions

Among Japanese university students, non-mutans streptococci bacteria in saliva were associated with increased caries in this 3-year prospective cohort study. However, there were no significant differences in salivary microbiome diversity between increased and non-increased groups.

## Figures and Tables

**Table 1:** Oral condition and health behaviors in total and selected participants at baseline.

Variable			Total Participants	Selected Participants	*p*-Value ^3^
			(*n* = 293)	(*n* = 55)	
Baseline	Gender	Male/Female	149/144 (50.9/49.1) ^1^	17/38 (30.9/69.1)	0.006
	Age (year)		18.22 ± 0.76 ^2^	18.24 ± 0.47	0.378
	Number of teeth present		28.49 ± 1.43	28.42 ± 1.49	0.699
	DMFT score		1.00 ± 2.01	1.44 ± 2.79	0.459
	DI-S		0.41 ± 0.37	0.33 ± 0.32	0.363
	Daily frequency of tooth brushing	1	55 (18.8)	9 (16.4)	0.914
		2	212 (72.4)	41 (74.5)	
		3	26 (8.9)	5 (9.1)	
	Use of dental floss	yes	20 (6.8)	5 (9.1)	0.551
	Regular dental check-ups	yes	36 (12.3)	13 (23.6)	0.034
Follow-up	Number of teeth present		29.2 ± 1.77	28.9 ± 1.78	0.353
	DMFT score		1.70 ± 2.99	1.82 ± 2.97	0.477
	DI-S		0.81 ± 2.49	0.31 ± 0.36	0.037
	Daily frequency of tooth brushing	1	45 (15.4)	8 (14.5)	0.323
		2	220 (75.1)	38 (69.1)	
		3	28 (9.6)	9 (16.4)	
	Use of dental floss	yes	45 (15.4)	12 (21.8)	0.237
	Regular dental check-ups	yes	41 (14.0)	11 (20.0)	0.301
	Current smoker	yes	9 (3.07)	2 (3.36)	
	Use of fluoride containing paste	yes	155 (52.9)	28 (50.9)	0.883
	Knowledge of the effectiveness of fluoride	yes	250 (85.3)	46 (83.6)	0.686
	Frequency of sweet intake (daily)	0	53 (18.1)	8 (14.5)	0.295
		1	163 (55.6)	37 (67.3)	
		2	56 (19.1)	9 (16.4)	
		3	21 (7.2)	1 (1.8)	

DMFT, decayed, missing, and filled teeth score; DI-S, debris index-simplified index; ^1^ Data are expressed as *n* (%); ^2^ Data are expressed as mean ± standard deviation; ^3^ Fisher’s exact test or Mann–Whitney U test.

**Table 2:** Differences in dental caries-related variables between increased and non-increased groups.

Variable			Total Participants(*n* = 293)			Selected Participants(*n* = 55)		
			Non-Increased	Increased	*p*-Value ^3^	Non-Increased	Increased	*p*-Value
			(*n* = 220)	(*n* = 73)		(*n* = 41)	(*n* = 14)	
Baseline	Number of teeth present		28.37 ± 0.37 ^1^	28.82 ± 1.65	0.017	28.27 ± 1.28	28.86 ± 1.95	0.127
	DMFT score		0.78 ± 0.12	1.68 ± 2.35	<0.001	1.32 ± 2.64	1.79 ± 3.28	0.71
	DI-S		0.38 ± 0.35	0.52 ± 0.42	0.008	0.33 ± 0.29	0.43 ± 0.41	0.619
	Daily frequency of tooth brushing	1	38 (17.3) ^2^	17 (23.3)	0.452	8 (19.5)	1 (7.1)	0.504
		2	161 (73.2)	51 (69.9)		29 (65.9)	12 (85.7)	
		3	21 (9.5)	5 (6.8)		4 (9.8)	1 (7.1)	
	Use of dental floss	yes	15 (6.8)	5 (6.8)	0.993	2 (4.9)	3 (21.4)	0.063
	Regular dental check-ups	yes	23 (10.5)	13 (17.8)	0.097	9 (22)	4 (28.6)	0.615
Follow-up	Number of teeth present		29.22 ± 1.77	29.36 ± 1.82	0.323	28.78 ± 1.74	29.29 ± 1.93	0.371
	DMFT score		0.78 ± 0.12	4.26 ± 3.98	<0.001	1.32 ± 2.64	3.28 ± 3.49	<0.001
	DI-S		0.94 ± 2.87	0.43 ± 0.42	0.932	0.33 ± 0.37	0.27 ± 0.37	0.372
	Daily frequency of tooth brushing	1	12 (15.4)	34 (15.4)	0.522	7 (17.8)	1 (7.1)	0.522
		2	61 (78.2)	164 (73.9)		27 (65.9)	11 (78.6)	
		3	5 (6.4)	24 (10.8)		7 (17.1)	2 (14.3)	
	Use of dental floss	yes	30 (13.6)	15 (20.5)	0.156	8 (19.5)	4 (28.6)	0.479
	Regular dental check-ups	yes	29 (13.2)	12 (16.4)	0.487	8 (19.5)	3 (21.4)	0.877
	Current smoker	yes	7 (3.2)	2 (2.7)	0.982	1 (2.4)	1 (7.1)	0.448
	Use of fluoride containing paste	yes	116 (52.7)	39 (53.4)	0.918	18 (43.9)	10 (71.4)	0.075
	Knowledge of the effectiveness of fluoride	yes	182 (82.7)	68 (93.2)	0.029	34 (82.9)	12 (85.7)	0.808
	Frequency of sweet intake (daily)	0	41 (18.6)	12 (16.4)	0.769	7 (17.1)	1 (7.1)	0.693
		1	124 (56.4)	39 (53.4)		29 (63.4)	11 (78.6)	
		2	39 (17.7)	17 (23.3)		7 (17.1)	2 (14.3)	
		3	16 (7.3)	5 (6.8)		1 (2.4)	0 (0.0)	

The increased group was defined as ΔDMFT > 0 during the 3-year follow-up period; The non-increased group was defined as ΔDMFT = 0 during the 3-year follow-up period; DMFT: decayed, missing, and filled teeth; DI-S, debris index-simplified. ^1^ Data are expressed as mean ± standard deviation. ^2^ Data are expressed as *n* (%). ^3^ Fisher’s exact test or Mann–Whitney U test.

**Table 3:** Comparison of relative abundances of bacteria between increased and non-increased groups.

Taxonomy Level		Non-Increased	Increased	*p*-Value ^2^
		(*n* = 41)	(*n* = 14)	
Phylum	*Proteobacteria*	10.5 ±7.8 ^1^	12.2 ± 4.3	0.029
Family	*Prevotellaceae*	1.6 ± 1.6	2.6 ± 2	0.007
Genera	*Alloprevotella*	1.6 ± 1.6	2.6 ± 2	0.007
	*Dialister*	0.2 ± 0.3	0.3 ± 0.5	0.039
	*Actinobaculum*	0.1 ± 0.1	0.1 ± 0.1	0.008
Species	*Neisseria sicca; n. mucosa*	2.3 ± 2.8	3 ± 2.5	0.042
	*Alloprevotella sp.*	1.1 ± 1.3	1.7 ± 1.7	0.009
	*Dialister invisus*	0.1 ± 0.3	0.3 ± 0.5	0.037
	*Cardiobacterium hominis*	0.3 ± 0.7	0.4 ± 0.6	0.037
	*Acinetobacter sp.*	0.3 ± 0.5	0.5 ± 0.5	0.001
	*GN02 [G-1]*	0.1 ± 0.3	0.2 ± 0.3	0.031
	*Neisseria elongata*	0.4 ± 0.8	0.7 ± 1	0.034
	*Actinomyces graevenitzii*	0.1 ± 0.1	0.1 ± 0.2	0.024
	*Anaerolineae [G-1]*	0.1 ± 0.1	0.2 ± 0.2	0.032
	*Dialister pneumosintes*	0.1 ± 0.1	0.1 ± 0.2	0.015
	*Haemophilus haemolyticus*	0.1 ± 0.2	0.2 ± 0.4	0.019
	*Actinobaculum sp.*	0.1 ± 0.1	0.1 ± 0.1	0.008
	*Corynebacterium matruchotii*	0.1 ± 0.1	0.1 ± 0.1	0.042
	*Prevotella pleuritidis*	0.1 ± 0.1	0.1 ± 0.2	0.014
	*Neisseria sp.*	0.1 ± 0.1	0.1 ± 0.1	0.021

^1^ Data are expressed as mean ± standard deviation; ^2^ Mann–Whitney U test.
